# Advanced Preoperative Imaging in Macula-Off Rhegmatogenous Retinal Detachment: Emerging Diagnostic and Prognostic Insights for Clinical Management

**DOI:** 10.3390/diagnostics16111581

**Published:** 2026-05-22

**Authors:** Lorenzo Motta, Rodolfo Mastropasqua, Michele Cillis, Giulia Craighero, Nicola Sereni, Corina De Santis, Alberto Quarta, Aldo Gelso, Giuseppe Lo Giudice, Claudio Iovino

**Affiliations:** 1 Department of Ophthalmology, School of Medicine, University of Padua, 35121 Padua, Italy; drlorenzomotta@gmail.com (L.M.); michelecillis.med@gmail.com (M.C.); giuliacr98@gmail.com (G.C.); 2 Department of Neurosciences, Imaging and Clinical Sciences, University “G. d’Annunzio” Chieti-Pescara, 66100 Chieti, Italy; rodolfo.mastropasqua@gmail.com (R.M.); corinadesantis@gmail.com (C.D.S.); alberto.quarta.96@gmail.com (A.Q.); 3 ULSS2 Marca Trevigiana, 31100 Treviso, Italy; nicola.sereni@aulss2.veneto.it; 4 Villa Dei Fiori, Acerra, 80011 Naples, Italy; a.gelso@libero.it; 5 U.O.C of Ophthalmology, “Ospedali Riuniti Padova Sud—ULSS 6”, 35121 Padua, Italy; gvofta@gmail.com

**Keywords:** artificial intelligence, macula-off retinal detachment, optical coherence tomography, preoperative imaging, retinal biomarkers, rhegmatogenous retinal detachment, ultra-widefield imaging

## Abstract

Retinal detachment (RD) is a potentially sight-threatening condition that requires timely diagnosis and appropriate surgical management. In macula-off rhegmatogenous retinal detachment (RRD), visual recovery after successful reattachment remains highly variable, highlighting the need for reliable preoperative prognostic markers. This study focuses on the contribution of advanced retinal imaging to the preoperative assessment of macula-off RRD, summarizing current evidence on imaging-derived biomarkers associated with disease severity and postoperative functional outcome. In this narrative review, we analyze studies employing spectral-domain and swept-source optical coherence tomography (SD-OCT and SS-OCT), OCT angiography (OCT-A), and adaptive optics OCT (AO-OCT) to characterize microstructural and microvascular retinal alterations. Emerging approaches, including ultra-widefield OCT (UWF-OCT) and artificial intelligence-based image analysis, are also discussed for their potential role in refining diagnosis, supporting surgical planning, and improving prognostic stratification. While several imaging parameters appear promising, their prognostic value is not yet fully realized. Further prospective studies are required to validate clinically meaningful imaging biomarkers and to integrate advanced imaging into routine preoperative decision-making for macula-off RRD.

## 1. Introduction

Retinal detachment (RD) represents a potentially sight-threatening condition which requires rapid clinical assessment and correct treatment in order to avoid irreversible legal blindness. This pathological entity is characterized by the separation of the neurosensory retina from the underlying retinal pigment epithelium (RPE). RD occurrence varies, but globally, it affects roughly 6 to 18 patients per 100.000 every year, with demographic and geographic differences and increasing incidence in recent decades [1,2].

The prevailing form is rhegmatogenous RD (RRD), due to the presence of a retinal break, “rhegma” in ancient Greek, that enables the entrance of liquified vitreous under the neurosensory retina, subsequently splitting the layers [3]. However, there are other forms of retinal detachment, such as exudative and tractional, which present with different pathophysiological mechanisms, that are not discussed in this article [4,5]. It is imperative, in clinical assessment and for treatment purposes, to consider the extension of the RD and the possible involvement of the macula, the retinal region containing the fovea, which is essential for centralized, detailed vision. RRD is therefore classified into “macula-on” and “macula-off” retinal detachment. This review focuses on “macula-off” RRD, which, despite early treatment and innovative surgical techniques, is associated with challenging treatment approaches and poorer visual prognosis when compared to macula-on RRD [6,7].

In about 90% of cases, retinal reattachment is achieved, using diverse techniques which include: scleral buckling (SB), pars plana vitrectomy (PPV) or a combination of these [6,7]. Postoperative functional outcomes, however, are variable. This is due to numerous different preoperative and postoperative factors, such as baseline best-corrected visual acuity (BCVA), macula involvement, the extent of the RD, the onset and duration of preoperative symptoms and vitreous traction [8,9].

When it comes to macula-off RD, due to the anatomical extent of the macular region and this being a timing-dependent condition, prompt clinical assessment plays a crucial role in reducing the risk of irreversible vision loss and establishing the correct surgical management [10,11,12].

Traditional clinical examination utilizing the BCVA, Amsler test and fundoscopy provide limited qualitative information. Modern advanced retinal imaging techniques, in particular spectral-domain and swept-source optical coherence tomography (SD-OCT and SS-OCT), are the standard in daily clinical practice, adding detailed quantitative microstructural information during preoperative evaluation, with multiple parameters, some with postoperative prognostic value [13,14].

Additionally, optical coherence tomography angiography (OCT-A) aims to further clarify microvascular elements to define the pathogenesis of the disease. Quantitative and qualitative OCTA metrics allow the analysis of retinal and choroidal alterations induced by RRD [15,16]. Adaptive optics OCT (AO-OCT) provides more detailed data regarding the disrupted cytoarchitecture of photoreceptors in preoperative and postoperative settings, useful in defining functional prognosis [17].

Other innovative imaging modalities such as ultra-widefield imaging including optical coherence tomography (UWF-OCT), with the assistance of artificial intelligence, may help guide surgical planning and functional prognosis in the clinical setting. In this narrative review, we aim to analyze and summarize all the advantages and limitations of advanced preoperative imaging in macula-off RRD.

## 2. Materials and Methods: Search Strategy Design

We conducted an exploration of the recent medical literature utilizing Pubmed, Google Scholar, and Scopus, researching significant studies that address advanced imaging techniques employed in evaluating RRD, focusing on “macula-off” RRD. The search included articles published in English up to December 2025, inluding both recent and relevant earlier studies. Keywords searched were combined to find significant articles that employed modern imaging modalities to analyze macula-off RRD. Researched words were “SD-OCT”, “SS-OCT”, “OCT-angiography”, “ultra widefield OCT” “adaptive optics-OCT”, “preoperative imaging in RD” and “artificial intelligence in RD”. We excluded other forms of RD (e.g., tractional or exudative) and other imaging modalities from the analysis. We included case-series, retrospective, and prospective studies, randomized clinical trials, and meta-analyses addressing imaging findings in macula-off RRD. Data extraction was performed qualitatively, focusing on the main imaging biomarkers, their diagnostic role, and their potential prognostic implications. This review of the literature has been intended to be narrative.

## 3. Advanced Imaging Techniques in the Preoperative Evaluation of Macula-Off RRD

### 3.1. Spectral-Domain OCT and Swept-Source OCT

SD-OCT is a high-resolution, non-invasive medical imaging technique with increased axial resolution and high scan speed. It captures cross-sectional images of the retina, improving sensitivity and specificity of microstructure analysis [13,14]. By comparison, the SS-OCT gains a deeper-penetration analysis, providing a histological-like, single-layer definition of retina morphology [13,14,17]. Both approaches are employed in preoperative evaluation of RD. Certain studies emphasize the role of these imaging tools in defining a classification of RRD, considering the macula and fovea status, which is a predictive factor of postoperative visual functions [13,14,17].

Specifically, Klaas et al. developed a potential classification, defining five grades (G1–G5) of macula-involving (MIRD) and central-involving retinal detachment (CIRD), adhering to the original ETDRS nomenclature [18]. If CIRD was limited to three outer ETDRS quadrants (G4), the mean BCVA was better compared to CIRD involving all four ETDRS quadrants (G5). Multivariate regression analysis demonstrates that a lower grade of detachment and lower extent of cystoid edema were both associated with better postoperative function [18]. These results are in line with other studies and correlate with the disruption of outer retinal layers and altered photoreceptor functionality [19,20]. However, Poulsen et al., in a prospective study that included macula-off RRD, reported that eyes with less microstructural alterations were associated with better visual outcomes in comparison to eyes with macular detachment with altered intraretinal appearance [21].

As support, Mané and colleagues report data from preoperative SD-OCT, demonstrating that a shallow RD involving the foveal center in macula-off RD had similar functional results to macula-on RD, probably due to preoperative reduced duration of RD and moderate loss of visual acuity [22].

There are other factors which play a role in functional outcomes that need to be considered. These include: age, onset and duration of symptoms, timing of surgical RD repair, surgical technique employed and quality of surgical abilities. Baseline visual acuity and timing to surgery are considered good predictors of visual outcomes, as demonstrated by Felfeli et al. and Chatziralli et al. [23,24]. Low visual acuity at baseline is associated with poorer postoperative functional outcomes, and delayed surgery is associated with alterations in outer retinal and photoreceptor functionality [23,24].

#### OCT-Derived Potential Biomarkers

In clinical management of macula-off RRD, multiple studies have attempted to examine emerging parameters from SD-OCT and SS-OCT in preoperative settings that may have a prognostic role, predicting visual function recovery [25]. OCT biomarkers are summarized in Table 1. Certain studies explored quantitative assessment of subretinal fluid volume and subsequently the height of retinal detachment (HRD) at the level of the fovea [18,26].

This biomarker has been reported by multiple studies as a potential predictor of postoperative visual function: higher foveal detachment height was correlated with low preoperative and postoperative functional outcomes [18,25,26]. However, a recent meta-analysis by Murtaza et al. found weak correlation between HRD and postoperative visual function [27].

Other studies have analyzed a possible association between retinal thickness (RT) and postoperative visual function [18,19,28]. Regarding central macular thickness (CMT), there is no clear relationship to postoperative visual acuity and further studies are needed to address this topic. However, Gharbiya et al. described a statistically significant relationship between outer nuclear layer (ONL) thickness and BCVA [29]. It was shown that eyes with photoreceptor layer disruption ≥ 200 μm had a significantly worse postoperative BCVA than those with less extensive disruption < 200 μm [29].

Subretinal hyperreflective points (HRPs) detected on OCT may represent inflammatory response by intraretinal cells or aggregates related to retinal injury.

In a retrospective study by Savastano et al., HRPs have been hypothesized to have a negative impact on postoperative visual function along with foveal detachment. The independent predictive value of HRPs in postoperative visual prognosis, however, is still debated, and further research is needed [30].

Russell and colleagues similarly reported that in RRD case-series, evidence on OCT of HRPs in the ellipsoid/interdigitation layers may help in dating the RD and understanding the distribution of the subretinal fluid and subsequently localizing retinal breaks [31].

Intraretinal cystic cavities (ICCs) in macula-off RRD eyes, described in multiple studies, are often associated with poorer prognosis, possibly indicating more severe retinal layer disruption. Preoperative ICCs extended in multiple retinal layers are associated with poor postoperative visual acuity due to diffused alterations and disruption of the blood–retinal barrier. Localized ICCs are not significantly correlated with postoperative visual acuity [27].

The integrity of the ellipsoid zone (EZ) and external limiting membrane (ELM) has been recognized as a predictor of postoperative visual function. Eyes with preoperative continuity of EZ and ELM have significantly better postoperative visual acuity [32]. Guan et al. reported that discontinuity of EZ was significantly associated with worse postoperative visual acuity [28]. Similarly, a recent study by Sassen et al. supports the relevance of EZ reflectivity (rEZR), a novel SD-OCT imaging biomarker for photoreceptor integrity; higher rEZR values were significantly associated with better BCVA and scleral buckling [33]. Moreover, a retrospective study by Vidal Olivier et al. supports the value of rEZR as a biomarker of photoreceptor metabolic recovery, and alterations in EZ and ELM are correlated with poor visual recovery [34].

Recently, bacillary layer detachment (BALAD) has been found in about one-fourth of cases with macula-off RRD [35]. BALAD has been defined as a characteristic intraphotoreceptor separation within the myoid zone, appearing on OCT as hyporeflective space beneath the ELM. Its onset has been attributed to tractional forces on the outer retina [36]. The presence of BALAD in macula-off RRD has been associated with poor visual outcomes after surgery and an increased risk of full-thickness macular hole after surgery [35,36].

Some authors investigated the role of outer retinal corrugations (ORCs), defined as multiple, consecutive high-frequency undulations evident at the level of the outer retina. In the study of Nagpal and colleagues, ORCs were associated with poor postoperative visual outcomes [37]; in other studies, this parameter was associated with alteration of reflectivity in the photoreceptor layer and the EZ [4,14,38]. Up for discussion is whether ORCs are the precursor of outer retinal folds observed in postoperative follow-up [35].

**Figure 1:**
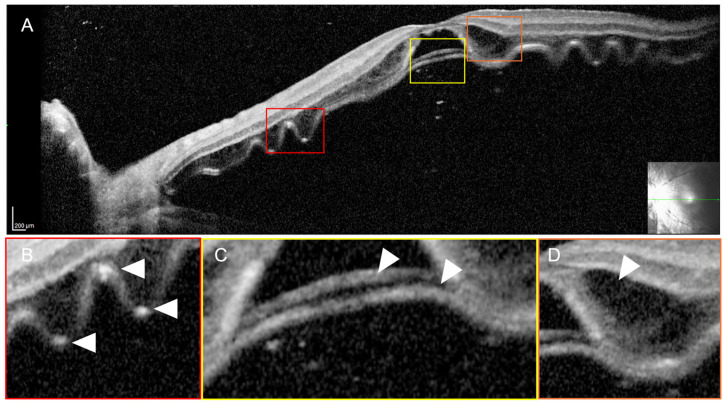
Spectral-domain optical coherence tomography B-scan of macula-off retinal detachment (**A**) showing outer retina corrugations (red square, (**B**)), bacillary layer detachment (yellow square, (**C**)), and intraretinal cystic cavities (orange square, (**D**)).

Figure 1 shows several OCT biomarkers including ORCs, BALAD and ICCs.

**Figure 2:**
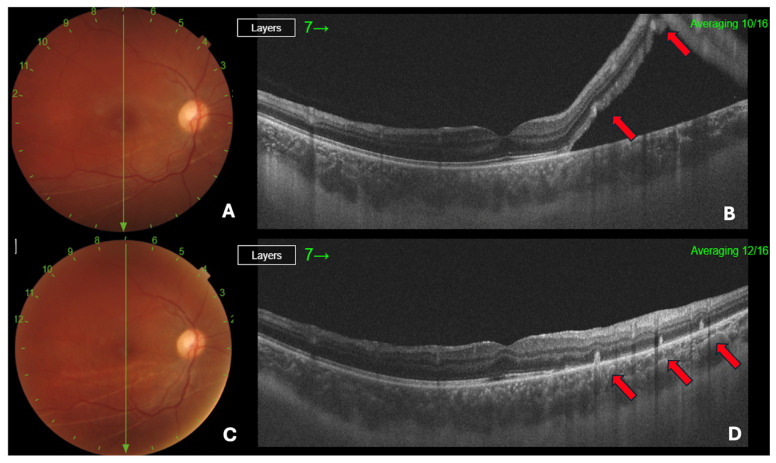
(**A**,**B**) Color fundus picture (CFP) and swept-source optical coherence tomography (SS-OCT) of preoperative inferior retinal detachment. The red arrows show the outer retinal folds (ORFs). (**C**,**D**) CFP and SS-OCT after pars plana vitrectomy show a reattached retina with persisting ORFs.

Besides ORCs, outer retinal undulations (ORUs) are a debated biomarker, as described in the retrospective study by Yeo et al. [19]. They may correlate with the duration of the RD; in particular, younger patients and those with early RRD were seen to have a higher incidence of outer retinal undulations, but there was a statistically significant relationship with postoperative visual outcomes [19]. Postoperative outer retinal folds (ORFs) have recently been considered an important anatomic factor affecting visual function in RRD surgery. ORFs appear on OCT scans as small, hyperreflective lesions extending obliquely or vertically into the outer nuclear layer (Figure 2).

The onset of ORFs has been reported to range between 34% and 42% after PPV, and although they do not have a negative impact on postoperative VA, ORFs are associated with metamorphopsia [39,40].

OCT features may be implemented and applied with other purposes—Martins Melo and colleagues proposed an innovative RRD staging system based on different imaging features accessed with SS-OCT [41]:

- Stage 1: separation of the neurosensory retina from the RPE.
- Stage 2: thickening of the photoreceptor layer.
- Stage 3: formation of ORCs, divided into
  ○ 3a: Low-frequency ORCs;
  ○ 3b: High-frequency ORCs.
- Stage 4: progressive loss of ORC definition with concurrent thickening of the photoreceptor layer.
- Stage 5: partial (“moth-eaten”) or complete loss of photoreceptors.

All OCT features and their prognostic consequences are summarized in Table 1.

SD-OCT and SS-OCT are useful imaging techniques in clinical management of macula-off RD, and multiple parameters and biomarkers may have prognostic value; however, surgical factors influence the evolution and the impact of these biomarkers in successful functional outcomes. More studies are needed to further clarify these topics.

### 3.2. OCT Angiography

OCT-A is an innovative imaging tool based on the principle of diffractive particle motion detection delineating retinal and choroidal vascularization that may be employed in the study of numerous retinal diseases [42]. From a multimodal imaging perspective, this modality acts as an adjunctive imaging technique with conventional dye angiography. Numerous parameters and metrics have been discovered, such as the foveal avascular zone (FAZ), foveal and parafoveal vessel density (VD) of retinal plexuses, and others, applied to study pathophysiological information in multiple retinal and choroidal disorders, such as diabetic retinopathy, age-related macular degeneration, and even RD [43,44].

OCT-A’s role in the preoperative management of macula-off RRD must be defined, and the use of this tool in macula-off RD is challenging due to motion artifacts, media opacities and segmentation errors. Several studies report changes in some OCT-A parameters (FAZ area, capillary density) after surgical repair. A clear relationship with postoperative visual function and the predictive role of these biomarkers in visual prognosis, however, are lacking [43,44,45]. Some authors found an increase in the FAZ and a reduction in VD, in superficial and/or deep capillary plexuses, with heterogeneous results, in eyes undergoing RD surgical repair in comparison with normal eyes [44,45,46,47,48].

Despite this insightful data, further studies investigating the role of OCT-A in macula-off RRD are needed to define preoperative biomarkers or microvascular changes with a significant predictive prognostic value and to aid in precise surgical decisions for every patient.

### 3.3. Ultra-Widefield Color Fundus Picture and Fundus Autofluorescence

Ultra-widefield (UWF) color fundus photography has significantly expanded the ability to document and evaluate peripheral retinal pathology compared to conventional 30–50° imaging systems. By capturing up to 200° of the retina in a single image, UWF imaging allows improved visualization of the periphery, facilitating detection of various retinal conditions, such as the full extent of RRD, which may influence surgical planning and management decisions [49].

Clinical studies have demonstrated that UWF imaging enhances identification of peripheral lesions which may be missed on standard photography, thereby improving diagnostic sensitivity and documentation in both medical and surgical retina practice [50]. Additionally, UWF systems provide rapid, noncontact acquisition, which is particularly advantageous in patients with limited cooperation or media opacity. However, there are limitations, including peripheral image distortion, due to the curved retinal surface and variable magnification toward the edges of the image, which may affect lesion size estimation and localization [51]. Despite these constraints, UWF color photography represents a valuable adjunct to dilated fundus examination and multimodal imaging, contributing to comprehensive assessment of peripheral retinal disease.

A study by Zuk et al. shows that traditional peripheral retinal evaluation with dilated examination or ultra-widefield color photography lacks cross-sectional detail of vitreoretinal and microstructural changes [52]. They combined UWF imaging with swept-source OCT to improve visualization and characterization of peripheral lesions. Even though some limits remain in optical artifacts and reduced sensitivity in far anterior regions, this technique might be taken into consideration and studied more [52].

**Figure 3:**
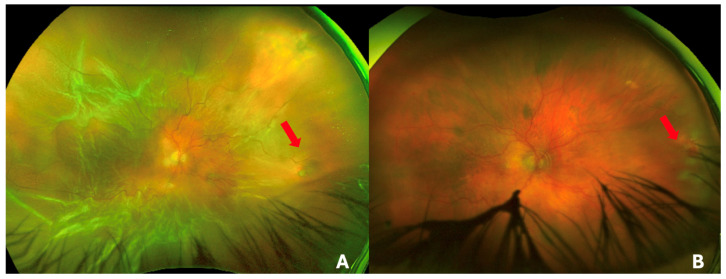
Ultra-widefield fundus picture (UWFP) images of a patient with total rhegmatogenous retinal detachment (RRD). (**A**) Preoperative UWFP assessment clearly showing the extension of the RD in all quadrants and the retinal break in the temporal nasal field (red arrow). (**B**) Postoperative UWFP showing a flat retina with laser scars surrounding the nasal retinal break and silicon oil in the vitreous chamber.

A study by Nadelmann and colleagues reports the use of Ultra-Widefield Autofluorescence (UWA) in the differential diagnosis of RRD and retinoschisis (RS): on UWF, eyes with RD appear bullous with a hyperreflective leading edge and homogeneous pattern in comparison with eyes with RS, conferring to this imaging tool an adjunctive and complementary utility in clinical assessment [53]. A representative case of UW color fundus photography is shown in Figure 3.

### 3.4. Ultra-Widefield OCT

With continuous implementation of imaging technology, emerging use of UWF-OCT through cross-sectional scan imaging of the entire retina, up to the peripheral retina, allows clinicians to obtain detailed images between 110 and 200° of view, from the anterior border of the vortex vein ampulla to the ora serrata, as described by the international widefield imaging study group [54].

This imaging modality is useful in clinical diagnosis and monitoring of numerous retinal diseases, including RRD. Multiple studies have demonstrated the enhanced diagnostic capability of UWF-OCT in detecting subtle peripheral retinal lesions [55,56,57]. In a large cohort study, the application of UWF-OCT revealed previously undetected significant peripheral lesions in 39% of cases, 31% of which were not identified with conventional OCT and slit-lamp examinations; moreover, in the same study, UWF-OCT aided in localizing retinal detachment, extension, and related characteristics, demonstrating its useful role in the clinical assessment of RD [58].

Other studies show that UWF-OCT provides significant anatomical information, helping clinicians in differential diagnosis with other pathological entities, directly influencing management decisions and treatment strategies [59,60,61].

Recently, in a cross-sectional study, Agarwal et al. expanded the detection of peripheral retinal lesions with UFW-OCT, predisposing and non-predisposing to RRD, providing information regarding tractional vitreous and suspected areas, helping clinicians in their management decisions [62]. Despite the high cost, limits of the techniques, motion artifacts, poor pupillary mydriasis, and limits, UWF-OCT is a valuable and emerging tool that may guide routine clinical management of macula-off RD, influencing surgical planning and functional prognosis. A summary of UW imaging modalities diagnostic role and clinical implications is provided in Table 2.

### 3.5. Emerging Imaging Techniques

#### Adaptive Optics OCT

AO in ophthalmology is applied to limit chromatic aberrations to obtain high-resolution images and study in vivo detailed tissue microstructure and, in particular, photoreceptor cytoarchitecture and fine cellular elements, with an axial resolution of 2 µm [17,63,64]. Some authors assessed microstructural changes in vivo in eyes affected by macula-off RD, reporting a complete disruption of retinal layer architecture, distortion of the cone mosaic and alteration in cone density [65]. In a prospective study by Reumueller et al., AO-OCT was utilized in macula-off RDs undergoing surgical repair [65]. During postoperative imaging, evidence of cone mosaic distortion was reported, even with visual improvement [65].

Recently, there has been evidence of changes in reflectivity of the EZ of photoreceptors, as demonstrated by Sassen et al. and sustained by Vidal-Oliver and colleagues, which may aid in augmenting knowledge of anatomical and functional recovery of photoreceptors in the context of RRD [33,34].

To date, there is no literature regarding the clinical applications of AO-OCT in preoperative management of macula-off RD. This is due to high-cost technologies, long durations of examination, artifacts and the nature of the pathology. Aside from this, AO-OCT is an innovative tool to monitor postoperative functional recovery, and it may help in understanding the pathophysiology of RD and metabolic changes in retinal cellular elements in a possible blindness-causing condition such as macula-off RRD.

### 3.6. Artificial Intelligence

AI is revolutionizing the clinical management of RRD by improving diagnostic accuracy, guiding preoperative decision-making, and potentially optimizing treatment outcomes [66].

AI models employing UWF fundus photography and OCT have been engineered to detect RD and differentiate macula status preoperatively [66,67].

Li et al. developed a pioneering deep learning system using over 24,000 UWF fundus images that precisely localized retinal detachment areas [67]. Similarly, Wang and colleagues report a deep learning model trained with 6000 ultrasound images and achieved an AOC of 0.998 with a sensitivity of 99.2% and a specificity of 99.8%, encouraging the role of this AI tool in supporting ophthalmologists’ clinical diagnosis [68]. The potential advantages of deep learning models in predicting the anatomical outcomes of RRD surgery were further investigated with positive outcomes [68,69,70,71].

Beyond detection capabilities, AI assists in anatomical and functional prognosis by integrating imaging biomarkers with clinical variables to predict postoperative visual outcomes after RRD repair. Deep learning fusion models combining UWF fundus images and OCT scans have predicted visual acuity outcomes with high accuracy [72,73].

Although AI systems do not replace retinal specialist expertise, they may serve as decision-support tools that liberate clinicians’ capacity to focus on personalized care and complex cases while improving timely treatment access.

## 4. Conclusions

Advanced imaging modalities in ophthalmology have revolutionized the clinical assessment of numerous retinal conditions, including macula-off RRD. The widespread use of SD-OCT and SS-OCT in different vitreoretinal disorders has enhanced the knowledge of pathophysiology of macula-off RRD, revealing multiple promising OCT parameters such as HRD, RT, ICCs, ORCs, ORUs, ORFs, HRPs, EZ/ELM disruption and rEZR, which may have predictive value in functional prognosis.

OCT-A is an interesting and emerging imaging tool which provides information about retinal and choroidal vasculature. Its role in preoperative assessment of macula-off RD is, however, still debated due to artifacts and limitations of the pathology itself. Microvascular OCT-A parameters, such as VD and FAZ, which increase after surgery, could provide additional information regarding retinal and choroidal recovery in postoperative settings.

Additionally, AO-OCT may be a complementary and adjunctive advanced diagnostic tool playing a role in photoreceptor anatomy and functionality, especially in research fields. Nevertheless, its use in daily clinical practice is not standard.

UWF color, FAF and OCT are useful imaging tools that assist clinicians in clinical diagnosis with a non-invasive approach, allowing an overall evaluation, especially useful in management of macula-off RRD. Furthermore, UWF imaging validity is promising in treatment planning, influencing technical and surgical decisions.

The emerging diffusion of AI and deep learning models in ophthalmology, as propitious instruments, will likely have a significant impact on the clinical evaluation of multiple retinal conditions, enhancing accuracy and efficacy in diagnosis along with prognostic values.

It is crucial to comprehend the limits and legal and ethical issues of these innovative tools in modern medicine practice. Further research is needed to better assess and manage macula-off RRD, a potential vision-threatening disease. Application of multimodal imaging, with development of deep learning models, may significantly help vitreoretinal surgeons in their daily work on diagnostic and therapeutic fronts.

## Figures and Tables

**Table 1:** Optical coherence tomography imaging features and functional correlations.

Imaging Feature	Imaging Modality	Prognostic Consequences
**Height of retinal detachment (HRD)**	SD-OCT/SS-OCT	Higher vertical foveal-RPE distance of the detached retina; may be weakly correlated with inferior functional outcomes.
**Retinal thickness and central macular thickness**	SD-OCT/SS-OCT	Variable functional outcomes and no clinical correlation in the literature; longer studies are needed.
**Subretinal hyperreflective points (HRPs)**	SD-OCT/SS-OCT	Independent predictive value of HRPs in postoperative visual prognosis is still debated; a possible negative functional correlation
**Intraretinal cystic cavities (ICCs)**	SD-OCT/SS-OCT	Focal ICCs may have slight effect on functional recovery; however, extensive involvement appears to predict worse VA.
**EZ and ELM integrity and relevance EZ reflectivity (rEZR)**	SD-OCT/SS-OCT	Discontinuity of EZ/ELM and alteration in EZ reflectivity (rEZR) are correlated with inferior functional outcomes, while integrity of both is associated with superior VA.
**Outer retinal corrugations (ORCs) and undulations (ORUs)**	SD-OCT/SS-OCT	Evidence of ORCs may be associated with inferior anatomical and functional outcomes, while the predictive value of ORUs is still debated.
**Outer retinal folds (ORFs)**	SD-OCT/SS-OCT	The onset of ORFs after surgery is associated with metamorphopsia.
**Bacillary layer detachment (BALAD)**	SD-OCT/SS-OCT	The onset of BALAD is associated with an increased risk of full-thickness macular hole after surgery.

**Table 2:** Ultra-widefield imaging modalities in macula-off RRD: diagnostic role and clinical implications.

Imaging Modality	Diagnostic Role	Advantages	Limitations
UWFP	Wide visualization (up to 200°) of RD extent and peripheral retinal breaks	Improves documentation and surgical planning; enhances detection of peripheral lesions	Peripheral distortion, variable magnification, lack of depth resolution
UWA	Hyperautofluorescent leading edge in RRD; Different autofluorescence patterns compared to RS	Differential diagnosis between RRD and RS	Limited specificity; influenced by metabolic and imaging factors
UWF-OCT	Cross-sectional imaging of peripheral retina; detection of vitreoretinal interface abnormalities and subtle peripheral lesions	Enhances diagnostic accuracy; supports surgical planning and lesion localization	Motion artifacts, high cost, reduced sensitivity in far anterior retina, pupillary mydriasis

## Data Availability

No new data were created or analyzed in this study. Data sharing is not applicable to this article.
